# Deep learning based low-cost high-accuracy diagnostic framework for dementia using comprehensive neuropsychological assessment profiles

**DOI:** 10.1186/s12877-018-0915-z

**Published:** 2018-10-03

**Authors:** Hyun-Soo Choi, Jin Yeong Choe, Hanjoo Kim, Ji Won Han, Yeon Kyung Chi, Kayoung Kim, Jongwoo Hong, Taehyun Kim, Tae Hui Kim, Sungroh Yoon, Ki Woong Kim

**Affiliations:** 1 Department of Electrical and Computer Engineering, Seoul National University, room 908 Bldg. 301, 1 Gwanak-ro, Gwanak-gu, Seoul, 08826 Korea; 2 Department of Brain and Cognitive Sciences, Seoul National University College of Natural Sciences, Seoul, Korea; 3 Department of Neuropsychiatry, Seoul National University Bundang Hospital, 82 Gumi-ro 173beon-gil, Bundang-gu, Gyeonggi, 13620 Korea; 4 Department of Psychiatry, Yonsei University Wonju Severance Christian Hospital, Wonju, Korea; 5 Department of Psychiatry, Seoul National University College of Medicine, Seoul, Korea

**Keywords:** Neuropsychological tests, Alzheimer disease, Dementia, Data mining, Deep learning

## Abstract

**Background:**

The conventional scores of the neuropsychological batteries are not fully optimized for diagnosing dementia despite their variety and abundance of information. To achieve low-cost high-accuracy diagnose performance for dementia using a neuropsychological battery, a novel framework is proposed using the response profiles of 2666 cognitively normal elderly individuals and 435 dementia patients who have participated in the Korean Longitudinal Study on Cognitive Aging and Dementia (KLOSCAD).

**Methods:**

The key idea of the proposed framework is to propose a cost-effective and precise two-stage classification procedure that employed Mini Mental Status Examination (MMSE) as a screening test and the KLOSCAD Neuropsychological Assessment Battery as a diagnostic test using deep learning. In addition, an evaluation procedure of redundant variables is introduced to prevent performance degradation. A missing data imputation method is also presented to increase the robustness by recovering information loss. The proposed deep neural networks (DNNs) architecture for the classification is validated through rigorous evaluation in comparison with various classifiers.

**Results:**

The k-nearest-neighbor imputation has been induced according to the proposed framework, and the proposed DNNs for two stage classification show the best accuracy compared to the other classifiers. Also, 49 redundant variables were removed, which improved diagnostic performance and suggested the potential of simplifying the assessment. Using this two-stage framework, we could get 8.06% higher diagnostic accuracy of dementia than MMSE alone and 64.13% less cost than KLOSCAD-N alone.

**Conclusion:**

The proposed framework could be applied to general dementia early detection programs to improve robustness, preciseness, and cost-effectiveness.

## Background

Neuropsychological assessments are essential for early diagnosing dementia and monitoring progression of dementia in both clinical and research settings, in advance of high-cost neuroimaging-based diagnoses such as magnetic resonance imaging (MRI) and positron emission tomography (PET). However, the abundant information of neuropsychological batteries other than their conventional total and/or subscale scores are not optimally employed in diagnosing and/or subclassifying dementia. [1–4]. In our previous works, we showed that a simple cognitive test such as a categorical verbal fluency test would provide an accurate diagnostic reference of dementia if we employed various response patterns in the test instead of its simple total score [5, 6]. In this regard, neuropsychological batteries that consist of multiple cognitive tests for evaluating multiple cognitive domains may improve the diagnostic accuracy of dementia considerably if we employ the response patterns of multiple cognitive tests together instead of conventional total and/or subscale scores.

Recently, data mining has shown remarkable performance in various fields including the medical fields [7]. Data mining is an interdisciplinary field of statistics, machine learning, visualization, database systems, and so on [8]. It focuses on discovering new meaningful information from a large dataset and provides us the information as understandable structure [8]. Especially, deep learning has recently emerged owing to big data and high-performance computing power. The deep learning is capable of exploiting the unknown structure from data to discover good representation. Thanks to this representation learning, the deep learning has overcome previous limitations of conventional approaches. Furthermore, the deep learning made great contributions to major advances in diverse fields including bioinformatics and medicine [9–15]. As we discussed ahead, although a large number of neuropsychological assessment data have been accumulated, hidden patterns in the data are not fully analyzed yet. To analyze the neuropsychological assessment data, the data mining using deep learning techniques can be utilized as a suitable approach. Mani et al. [16] first applied the data mining approach to neuropsychological assessment data, but simple classifiers were used to show the possibility of data mining application to neuropsychological data. Leighty [17] and Maroco et al. [18] provided the useful comparison on applications of multiple machine learning classifiers to neuropsychological assessment data, but these research studies did not consider variable redundancy, which may cause the performance degradation arising from the curse of dimensionality. Lemos [19] applied variable selection algorithms to overcome the curse of dimensionality, but the approach just removed the data with missing values, which may lead to loss of information.

In this paper, to develop a practical data mining framework overcoming the issues raised in the previous works, we propose a deep learning based low-cost and high-accuracy diagnostic framework of dementia with the response profiles of the Korean Longitudinal Study on Cognitive Aging and Dementia Neuropsychological Battery (KLOSCAD-N). The framework includes design procedures on missing data imputation, input variable selection, and cascaded classifier design for cost effective classification. First, in contrast to the previous works discarding the missing data samples which lead to information loss, we introduce a missing data imputation procedure to increase the accuracy and robustness in data analysis. Second, to maximize the diagnostic performance, a deep neural networks (DNNs) architecture are designed and validated in comparison with the other well-known classifiers. Third, to prevent a degradation of classification performance arising from the useless or redundant variables, we suggest a procedure to check the existence of useless or redundant variables and prune them. Fourth, we design a two-stage classifier to reduce time and cost for diagnosis using KLOSCAD-N and MMSE.

## Methods

**Fig. 1:**
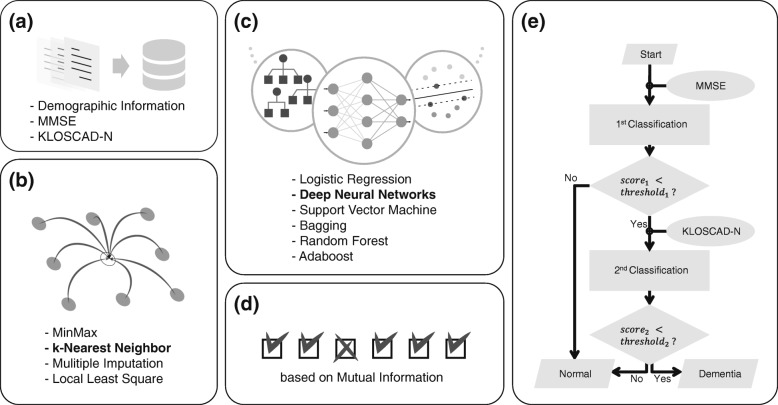
Overall scheme. The proposed diagnostic framework includes five steps. **a** Data Acquisition, **b** Missing Data Imputation, **c** Design and Validation of Classifier, **d** Input Variable Selection, **e** Two-stage Classification

Figure 1 depicts the overall scheme of the proposed diagnostic framework which includes five steps: (1) acquisition of KLOSCAD-N response profiles, (2) imputation of missing variables, (3) design of DNNs and validation by comparing with other classifiers, (4) input variable selection based on mutual information, and (5) design of two-stage classification scheme via the combination of MMSE and KLOSCAD-N. This study was approved by the institutional review board of Seoul National University of Bundang Hospital. The details of each step are provided in the following.

### Subjects

We analyzed the KLOSCAD-N response profiles of 2666 cognitively normal elderly (CNE) individuals and 435 dementia patients. The CNE individuals were the participants of the Korean Longitudinal Study on Cognitive Aging and Dementia (KLOSCAD), which is a community-based longitudinal study of cognitive aging and dementia of community-dwelling Korean elderly cohort [20]. The dementia patients were either participant of the KLOCSCAD or visitors to the 14 dementia clinics that participated in the KLOSCAD. All subjects were 60 years or older. We excluded subjects with major axis I psychiatric disorders, such as major depressive disorder, and those who had serious medical or neurological disorders that could affect cognitive functions. The demographic and clinical characteristics of the subjects are summarized in Table 1. The 20% of subjects were randomly chosen as a test dataset for evaluating the proposed framework. The test dataset was not used in any of training procedure. Using the remaining 80% of subjects as a train dataset, we carried out five-fold cross-validation for training and model selection.

**Table 1:** Characteristics of the subjects

	Controls	Dementia		Statistics	
		CDR=0.5	CDR=1	For *X*^2^	post *hoc*^*‡*^
Number	2666	189	246		
Age (years)	69.54±	75.01±	76.61±	174.927^∗∗∗^	*a*<*b*
	6.52^a^	7.23^b^	7.43^b^		
Sex	53.2	56.6	65.4	20.138^∗∗^	
(female, %)					
Education	9.57±	8.40±	6.61±	30.520^∗∗^	*a*>*b*>*c*
(years)	5.33^a^	5.75^b^	5.75^c^		

^∗∗∗^*p*<.001, ^∗∗^*p*<.01, ^*‡*^Games-Howell post hoc comparisons

a, b, c: the same letters indicate homogeneous groups

### Diagnostic Assessments

Research neuropsychiatrists evaluated each subject using a standardized clinical interview, physical and neurological examinations, and laboratory tests according to the protocol of the Korean version of the Consortium to Establish a Registry for Alzheimer’s Disease Assessment Packet (CERAD-K) [21] and the Mini International Neuropsychiatric Interview (MINI) version 5.0 [22]. When dementia was suspected, brain computerized tomography (CT) or magnetic resonance imaging (MRI) was also performed. The subjects diagnosed as having dementia according to the criteria of the fourth edition of the Diagnostic and Statistical Manual of Mental Disorders (DSM-IV) (American Psychiatric Association 1994) were enrolled in the dementia group. The global severity of dementia was determined according to the Clinical Dementia Rating (CDR) [23].

### Neuropsychological assessments

Trained research neuropsychologists who were blind to the diagnosis of the subjects administered the KLOSCAD-N to each subject. The KLOSCAD-N consists of the Korean version of the Consortium to Establish a Registry for Alzheimer’s Disease Assessment Neuropsychological Battery (CERAD-N) [21, 24], Digit Span Test (DST) [25], Frontal Assessment Battery (FAB) [26], and Executive Clock Drawing (CLOX) [27]. The CERAD-N consists of nine neuropsychological tests: Categorical Verbal Fluency Test (CVFT), 15-item Boston Naming Test (BNT15), MMSE, Word List Memory Test (WLMT), Constructional Praxis Test (CPT), Word List Recall Test (WLRT), Word List Recognition Test (WLRCT), Constructional Recall Test (CRT), and Trail Making Test A and B (TMT-A and TMT-B). Conventionally, test scores of the nine neuropsychological tests were used to ascertain the presence of cognitive impairment objectively in diagnosing dementia and monitor the progress of cognitive impairment objectively with advancing dementia.

### Missing data imputation

Inputs with missing values is unable to apply most of supervised machine learning models including deep learning. On the other hand, since the missing values often appear in neuropsychological tests, it is necessary to make up the missing values in order to apply the model to the subjects having the missing values. Among the 3101 samples of KLOSCAD-N response profiles, 75 have at least one missing value. Samples with one or two missing values are most frequent. CLOX1 and CLOX2 scores have the most frequent missing values. We have implemented four imputation methods: minimum-maximum (MinMax) imputation, k-nearest-neighbor (kNN) imputation [28], multiple imputations (MI) (Schafer 1999), and local least squares (LLS) imputation [29].

First, the MinMax imputation method is based on the assumption that the missing is caused by the subject’s deficiency. The missing values are imputed according to the correlation between variables and labels. If the correlation is positive (or negative), the missing value is imputed with the maximum (or minimum) value of the variable. Second, the kNN imputation method attributes the missing values using the information of other subjects with a similar pattern in that sense of the nearest neighbor. After finding k number of neighbors, the imputation value is computed by averaging the values of those neighbors. In this study, Euclidean distance is used, and k is set to 5 empirically via experiments. Third, the MI method provided by the SPSS software is the most popular method in statistics, which has been developed to solve a single imputation’s underestimating problem. The missing values are replaced by averaging a number of complete datasets which are estimated by the Monte Carlo technique. Each estimated complete dataset is imputed by linear regression. Lastly, the LLS imputation method shows the best performance for the missing value estimation on microarray data [30]. After finding the top k number of relevant genes (variables) using Pearson correlation, the target gene and its missing value are obtained by a linear combination of those relevant genes through solving a least squares problem.

Each method is evaluated in two ways: direct evaluation via error computation and indirect evaluation via classification performance. The direct evaluation is to compute an error between the original value and the imputed value. After we randomly generate artificial missing data from the complete data by considering the missing ratio in each variable, four kinds of imputation values for the artificial missing data are obtained through the four methods, respectively. The error between the original value and the estimated values is computed by matrix Euclidean norm. The indirect evaluation is to check a classification performance on imputed samples using the classifier trained with the complete data. By utilizing the four kinds of imputed samples generated by the four methods, respectively, we check which method shows the best classification performance by various classifiers.

### Constructing deep learning classifiers

Artificial neural network (ANN) is a computation model inspired by the biological brain. The hidden layer of ANN takes a role of feature extraction from input or lower hidden layer information. The responses in the hidden layer represent features extracted via a linear transformation of inputs and a nonlinear activation functions. The DNN is a kind of ANN with deep hidden layers between the input and output layers. The deep layers composite the features from lower layers hierarchically, and learn complex data by associative memorizing through connection weights [31].

**Fig. 2:**
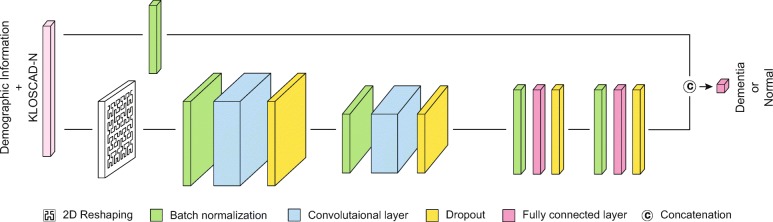
Architecture of proposed deep neural networks for KLOSCAD-N assessment and demographic information

To construct a promising diagnosis framework, we design the DNNs for MMSE and KLOSCAD-N respectively. Since MMSE is composed of only five dimension (four demographic variables and one MMSE total-score), the fully-connected network (FCN) is enough to cover this simple classification problem. For KLOSCAD-N, we construct a two dimensional convolutional neural network (2D-CNN) to achieve the best performance. As shown in Fig. 2, we cascade a fully-connected layer following the convolutional layers. Also skip connection [32] is utilized to explicitly feed low level features to the output layers. In addition, we reshape the input into 2D image-like form with the Hilbert space-filing curve [33] which has been successfully used for DNA sequence classification with CNN [34]. Hilbert curve, which is shown in Fig. 2, give a mapping 1D to 2D space that fairly well preserves locality. Since our data is a sequence of assessments followed by demographic information, continuity and clustering property of Hilbert curve would be appropriate for our data characteristics. To prevent an over-fitting, dropout [35], batch normalization [36] and early stop training technique is applied. In this study, the ratio of the negative label samples to the positive label samples is approximately 9:1 because the positive samples indicating the subjects of dementia are relatively rare compared to the negative samples indicating normal subjects. To solve this problem, the cost-sensitive loss is defined as (1) by multiplying a weight with the positive target.

1 $$  l_{c}(y_{i}, \hat{y}_{i}) = -w_{c} y_{i} \log \hat{y}_{i} - (1-y_{i}) \log (1-\hat{y}_{i}),  $$

where *y*_*i*_ is target value, $\hat {y}_{i}$ is predicted value, and *w*_*c*_=(# of positive)/(# of negative). To achieve the optimal architecture, we empirically evaluate the model with all combination of hyper-parameters as follows: the number of convolution layer: [ 1,4], the number of filters: 32,64,128, kernel size: [ 2,4], the number of fully connected layer: [ 1,4], and the number of hidden unit: 32,64,128.

In addition, empirical evaluations are conducted for other architectures of DNNs such as 1D-CNN, fully-connected networks (FCN). Also we compare with the transfer learning by adopting a pre-trained model (NasNet [37]) since NasNet is capable to handle low dimensional inputs unlike other networks for imagnet. Also we compare our classifier with six well-known classifiers: XGBoost [38], Adaboost [39], Random Forest [40], Bagging [41], SVM [42], and Logistic Regression [43]. Hyper-parameters are empirically established through greedy search. Each algorithm is implemented by calling the java object of libSVM [44] and Weka [45] in MATLAB. To evaluate the generalization of each classifier, a five-fold cross validation on train dataset is applied. The area under curve (AUC) is used as the main evaluation metric.

### Input variable selection

Since useless or redundant variables cause a degradation of classification performance due to a curse of dimensionality, it is necessary to check the existence of useless or redundant variables among KLOSCAD-N. Furthermore, by eliminating the redundant variables, the assessment time and monetary costs can be reduced. If there is a hierarchical property between variables, it is difficult to independently remove each variable. In this study, we thus do not consider subtotal variables that belong to the upper part of the hierarchical structure but use only the scores of the lowest-level variables. The relationships (or hierarchical properties) among the selected variables are then analyzed through the 2D-CNN.

For this purpose, we adopt the feature selection toolbox (FEAST) [46] which provides a computation toolbox of mutual information and other information theoretic functions. FEAST calculates the ranking of all variables by their contribution of information. In our work, we utilize eight functions in FEAST: MIM, MRMR, CMIM, JMI, DISR, CIFE, ICAP, and CONDRED (see [46], the paper of FEAST toolbox, for details of each function). The ranking information of the eight functions is combined to determine the final ranking of each variable in an ensemble manner. For each variable, the eight ranking scores are averaged. The averaged ranking score is used to determine the ranking order of each variable.

Let *S*_*i*_,*i*=1,...,*m* be the variable set containing *i* number of variables in ranking order. For example, *S*_1_ only includes the highest ranked variable, and *S*_5_ includes the variables from the first rank to the fifth rank. Then the classification performance is evaluated for each set *S*_*i*_, and the set with the maximum performance is denoted by *S*_*max*_. DeLong’s test [47] is a statistical nonparametric approach to check whether two area under curve (AUC) values are having significant different. If the p-value from the test is less than 0.05, this indicates that the two sets show significant differences in AUC performance. Conversely, if the p-value is greater than 0.05, it can be judged that there is no significant loss of AUC performance between the two sets. Since the goal is to select the set with the lowest number of variables without loss of performance, we finally choose the set with the smallest number of variables from *S*_*i*_ with p-value over 0.05.

### Two-stage classification

MMSE is the most popular screening test for dementia [20, 21, 48, 49]. MMSE is advantageous at low cost, but it is known to be less accurate than high-cost batteries such as KLOSCAD-N. Therefore, we propose a novel framework that combines the advantages of MMSE and KLOSCAD-N. In the first stage, MMSE is applied as a coarse screening test, and in the second stage, the KLOSCAD-N is administered for a fine diagnosis. If the candidate for KLOSCAD-N can be reduced through the first stage (MMSE) in advance without loss of diagnostic performance, a low-cost and high-performance diagnostic framework could be established.

The brief block diagram of the two-stage classification framework is shown in Fig. 1e. The suggested framework has been established using the DNNs which showed the best performance among the other classifier on each test in the classifier comparison step. The MMSE total-score and demographic information are utilized to decide the further execution of the second stage, KLOSCAD-N, or not. By changing the threshold on the first-stage decision score to pass the subjects to the second-stage, we compute the cost and accuracy of the two-stage classification framework with test dataset. The cost is defined as

2 $$ cost = n_{all} \times c_{M} + n_{2} \times c_{K},  $$

where *c*_*M*_ and *c*_*K*_ is the cost per single subject of MMSE and KLOSCAD-N respectively, *n*_*all*_ is the number of all subjects, and *n*_2_ is the number of subjects who need the second-stage. Based on Korean insurance fees, the cost of each assessment per subject is approximately 10 USD and 180 USD for MMSE and KLOSCAD-N, respectively. We determine the best threshold on the decision score which shows the lowest cost while the performance does not show loss of classification performance.

## Results

### Missing data imputation

As suggested in the “Missing data imputation” section, the four imputation methods were evaluated via two ways, and the best imputation method was chosen. The first evaluation result (Euclidian norm) which gives the error between the original value and the imputed value was 1438.5621 for MinMax, 196.2499 for kNN, 255.7012 for MI, and 245.9988 for LLS. kNN had the smallest Euclidean error, whereas MinMax had the largest error. In consequence, kNN was evaluated to reconstruct the missing variable with the most similar value to the original one. Table 2 shows the result of the second evaluation approach, where the validity of imputed data had been evaluated by the classification performance tested via six classifiers trained with the complete data. Every classifier, except SVM, showed the best performance on kNN-based imputed data, whereas SVM showed the best performance on LLS. According to the result, kNN imputation method is chosen as the best one for the completion of missing values in KLOSCAD-N.

**Table 2:** Classification performances on the imputed dataset indicated by the area under the receiver operator curve (AUC)

	Proposed DNNs	XGBoost	Logistic Regression	Random Forest	Adaboost	Bagging	Support Vector Machine
MinMax	0.9489	0.9506	0.9083	0.9405	0.9149	0.9334	0.8898
kNN	0.9603	0.9541	0.9356	0.9466	0.9444	0.9559	0.9321
MI	0.9586	0.9524	0.9312	0.9211	0.9184	0.9418	0.9347
LLS	0.9594	0.9471	0.9295	0.9343	0.9109	0.9339	0.9383

MinMax: minimum-maximum imputation, kNN: k nearest neighbor imputation, MI: multiple imputation, LLS: local least square imputation

### Classifier validation

As we mentioned in the “Constructing deep learning classifiers” section, hyper-parameters for every candidate model were searched via greedy search. The best FCN for MMSE is composed of one layer with 128 number of hidden units. The best 2D-CNN model for KLOSCAD-N is composed with two convolutional layers which contains 128 and 32 number of filters respectively with kernel size of 2, and two fully connected layers with 64 hidden units. Skip connection leads to a performance improvement over all structures. For 2D-CNN, our input reshaping method with Hilbert curve achieves higher performance than naïve reshaping method that simply stacks a sliced 1D input to form of 2D matrix (see the second column in Table 3).

**Table 3:** Classification performances of various deep neural network architectures on Mini Mental Status Exam (MMSE) and Korean Longitudinal Study on Cognitive Aging and Dementia Neuropsychological Battery (KLOSCAD-N) indicated by the area under the receiver operator curve (AUC) via five-cross validation on train dataset

		2D-CNN	2D-CNN Naïve	2D-CNN w/o SC	1D-CNN	1D-CNN w/o SC	FCN	FCN w/o SC	NasNet
MMSE^a^	mean	-	-	-	-	-	0.9702	0.9583	-
	std	-	-	-	-	-	0.0144	0.0139	-
KLOSCAD-N	mean	0.9863	0.9850	0.9782	0.9848	0.9805	0.9830	0.9771	0.9813
	std	0.0048	0.0058	0.0057	0.0053	0.0042	0.0060	0.0070	0.0046

^a^Since MMSE is composed with only five dimension (four demographic variables and one MMSE total-score, the other architecture are not applicable except FCN

Transfer learning with weights pretrained from imagenet (NasNet) has shown AUC value of 0.9813, which is smaller than those of the other networks trained with random initialization. This implies the pretrained information from imagenet datasets is not helpful to solve our problem. Table 3 shows the classification performance of various deep learning architectures from five-fold cross validation. For MMSE, the designed FCN in our work has AUC value of 0.9702. For KLOSCAD-N, the proposed architecture for 2D-CNN shows the best performance (AUC value of 0.9863) among all the candidate architectures.

Table 4 shows the classification performance of other type of classifiers. For both MMSE and KLOSCAD-N, the proposed DNNs show the best performance. It is known that the DNNs show inherently a good generalization capability, even its large number of parameters when trained with the sufficient number of train data samples. As a result, our dataset is enough to achieve reasonable performance for the both assessment using the designed DNNs.

**Table 4:** Comparative analysis with other conventional classifiers indicated by the area under the receiver operator curve (AUC) via five-cross validation on train dataset

		Proposed DNNs	XGBoost	AdaBoost	Random Forest	Bagging	Support Vector Machine	Logistic Regression
MMSE	mean	0.9702	0.9605	0.9573	0.9581	0.9631	0.9627	0.9642
	std	0.0144	0.0144	0.0171	0.0192	0.0169	0.0196	0.0171
KLOSCAD-N	mean	0.9863	0.9850	0.9774	0.9762	0.9724	0.9744	0.9807
	std	0.0048	0.0065	0.0107	0.0079	0.0069	0.0093	0.0080

Table 5 shows the comparative efficiency of the proposed two-stage classification in view of various metrics including the cost. As shown in the fourth and fifth columns, the existing works for KLOSCAD-N and MMSE do not show good performance relatively because they rely on the simple total score of KLOSCAD-N or MMSE. As shown in the first and third columns DNNs improves the accuracy with 2.90% for MMSE and 6.61% for KLOSCAD-N compared to the existing methods because it can utilize the hidden patterns of input variables (demographic information, subscale scores, and so on). As shown in the second column, the proposed two-stage classification framework shows the best efficiency through all evaluation metrics with a reasonable cost (Details are discussed in the following section on two stage classification).

**Table 5:** Comparative results of two-stage classification on test dataset

	KLOSCAD-N w/ DNNs	Proposed Two-stage Classification	MMSE w/ DNNs	KLOSCAD-N w/o DNNs	MMSE w/o DNNs
Accuracy (%)	92.74	92.90	87.74	86.13	84.84
AUC	0.9790	-^a^	0.9383	0.9349	0.9143
F1 Score	0.7805	0.7800	0.6667	0.6356	0.6179
Sensitivity	0.9287	0.9343	0.8780	0.8621	0.8736
Specificity	0.9195	0.8966	0.8736	0.8612	0.8443
Likelihood Ratio Plus	11.5425	9.0319	6.9446	6.2092	5.6097
Likelihood Ratio Minus	0.0775	0.0732	0.1396	0.1602	0.1498
Positive Predictive Value	0.5673	0.5064	0.4410	0.4136	0.3892
Negative Predictive Value	0.9913	0.9917	0.9844	0.9821	0.9833
Pre Test Odd	0.1136	0.1136	0.1136	0.1136	0.1136
Post Test Odd	1.3111	1.0259	0.7888	0.7053	0.6372
Post Test Probability	0.5673	0.5064	0.4410	0.4136	0.3892
Cost^b^	$111,600	$40,030	$6,200	$111,600	$6,200

^a^Since each stage provides their own probability, single AUC value can not be calculated

^b^Total cost for test dataset including 620 subjects

### Input variable selection

**Fig. 3:**
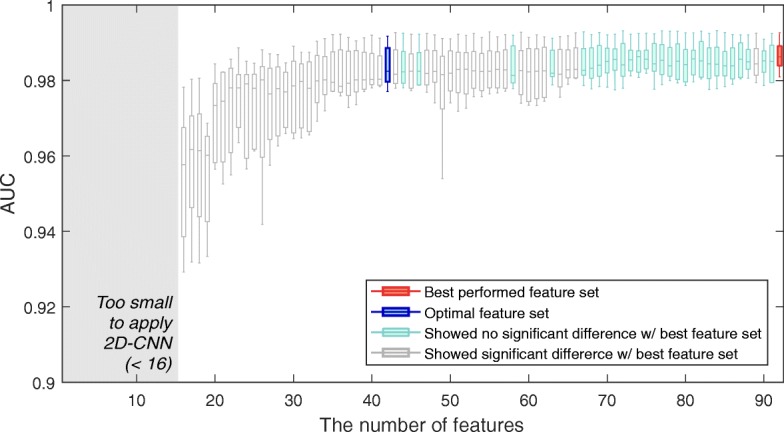
Dependency on the variables. Trends of the area under the receiver operator curve (AUC) as a function of the number of variables included in order from the highest ranging variable

The final rankings of 92 input variables were yielded through the ensemble of eight methods for feature selection provided in FEAST. The performances on the input variable sets, *S*_*i*_,*i*=1,...,92, are shown in Fig. 3. As shown in Fig. 3, the performance increases as the variables are added one by one in order from the highest-ranking variable, but the degree of increase lessens after 30 variables and becomes saturated after 43 variables. The best performance was achieved with 92 variables which are depicted as red boxplot in Fig. 3. Among *S*_*i*_, we removed the variable set (gray boxplot) that showed a significant difference (*p*<0.05 on DeLong’s test) with the best-performed variable set *S*_*max*_ (red boxplot). Among the remaining candidate variable set (blue boxplot and red boxplot), we chose the final variable set which contains the least number of variables. As a result, we could reduce the number of variables 92 to 43. The final variable set and variable ranking information is described in Table 6.

**Table 6:** Top 43 variables selected for classifying dementia from normal controls

Ranking	Variable description
1	Time to complete the Trail Making Test A
2	Retention index of Constructional Recall Test^a^
3	Age
4	Response bias index of the Word List Recognition Test^b^
5	Recency index of the Word List Memory Test^c^
6	Executive Clock Drawing Test (CLOX) 1 score
7	Consistency index of the Word List Memory Test^d^
8	Correct responses at the second quarter (15–30 s) in the Verbal Fluency Test
9	The number of repetitive recalls in trial 3 of the Word List Memory Test
10	Geriatric Depression Scale score
11	Cube recall score of the Constructional Recall Test
12	Clustering index of Verbal Fluency Test
13	Correct responses in the middle-frequency objects of the 15-item Boston Naming Test without cues
14	The number of correct recall in trial 2 of the Word List Memory Test
15	Digit Span Test Forward score
16	Years of education
17	Perceptual error index in the low-frequency objects of the 15-item Boston Naming Test
18	Ineffective switch index of the Verbal Fluency Test
19	Retention index of the Word List Recall Test^e^
20	Consistency index of the Word List Recall Test^f^
21	Primacy index of the Word List Memory Test^g^
22	Word List Recall Test score
23	Switch index of the Verbal Fluency Test^h^
24	The number of correct recall in trial 1 of the Word List Memory Test
25	Forward span of the Digit Span Test
26	Word List Recognition Test total score
27	Correct responses in the low-frequency objects of the 15-item Boston Naming Test with phonemic cues
28	Learning curve of the Word List Memory Test^i^
29	Digit Span Test Backward score
30	Correct responses at the last quarter (45–60 s) in the Verbal Fluency Test
31	Constructional Recognition Test score
32	Go-No-Go score of the Frontal Assessment Battery
33	The umber of correct recall in trial 3 of the Word List Memory Test
34	Correct responses in the high-frequency objects of the 15-item Boston Naming Test without cues
35	Correct responses at the first quarter (0–15 s) in the Verbal Fluency Test
36	’Do not know’ responses in the low-frequency objects of the 15-item Boston Naming Test
37	The number of intrusion errors in the Word List Recall Test
38	Intersecting rectangles recall score of the Constructional Recall Test
39	Recency index in trial 1 of the Word List Memory Test
40	Correct responses at the third quarter (30–45 s) in the Verbal Fluency Test
41	Backward span of the Digit Span Test
42	Diamond recall score of the Constructional Recall Test
43	Cube score of the Constructional Praxis Test

^a^(Constructional recall test score /constructional praxis test) ×100

^b^(False positive score −false negative score) /(false positive score+false negative score)

^c^(The number of recalled words among the last 3 words of the Word List Memory Test /Word List Memory Test score) ×100

^d^The sum of the numbers of words consistently recalled in between trial 1, trial 2 and trial 3 of the Word List Memory Test

^e^(Word List Recall Test total score/trial 3 score of Word List Memory Test) ×100

^f^(The number of words consistently recalled in the World List Recall Test among the recalled words in the Word List Memory Test) × 100

^g^(The number of recalled words among the first 3 words of the Word List Memory Test /Word List Memory Test score) ×100

^h^The number of switches between clusters during Verbal Fluency Test

^i^The number of recalled words in trial 3 of the Word List Memory Test - the number of recalled words in trial 1 of the Word List Memory Test

### Two-stage classifications

**Fig. 4:**
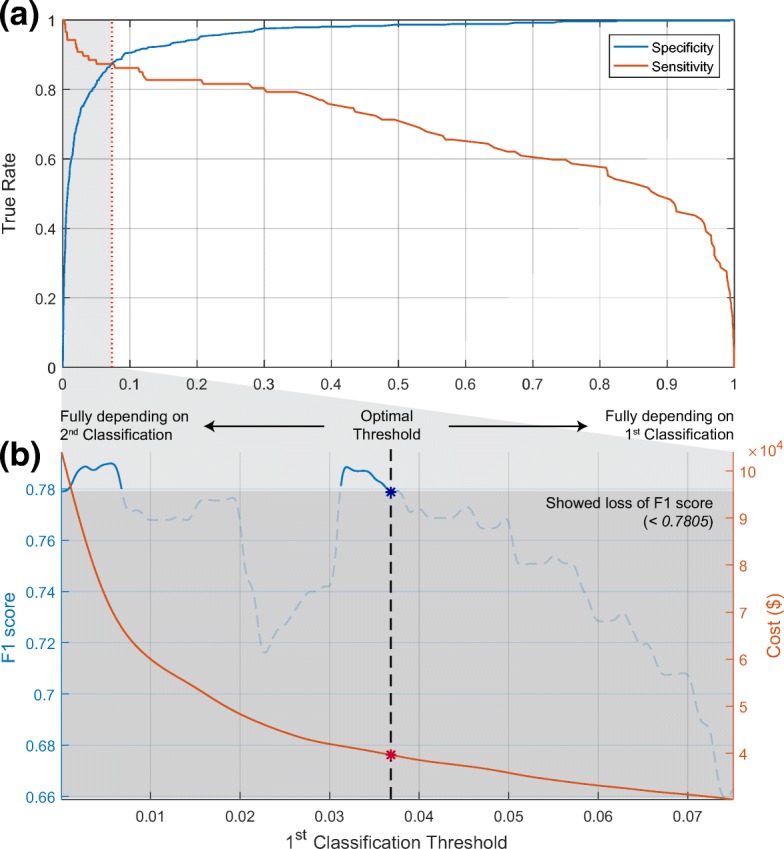
Dependency on the sweeping first classification threshold. Two-stage classification performance trends as function of a sweeping threshold of deep neural networks (DNNs) with MMSE for the second-stage diagnosis with Korean Longitudinal Study on Cognitive Aging and Dementia Neuropsychological Battery. **a** Equal error rate (EER) curve on DNNs for MMSE. **b** Empirically estimated performance and cost on test dataset. When first-stage classification threshold values is 0.0362, cost is minimized without any loss on performance (f1 score)

Accordingly, at two-stage classification, performance and cost were evaluated by changing the threshold of the first stage classification on MMSE to pass subjects to the second stage (KLOSCAD-N). The results are shown in Fig. 4. Figure 4a shows a value of sensitivity and specificity as a function of threshold on the first classification. It is noted that the two curves meet at the threshold of 0.075, and the point is referred to as equal error rate (EER). Figure 4b shows the trends of performance and cost in the threshold range [0,0.075]. As shown in Fig. 4b, the higher threshold (fewer subjects take KLOSCAD-N) leads to the less performance and cost. On certain the threshold, f1 scores are smaller than that of when the threshold is zero. In conclusion, at threshold equal to 0.0362, the proposed framework save as much as cost without loss of performance. The second column in Table 5 is the final performance of the proposed two-stage classification. As a result of the proposed combination of MMSE and KLOSCAD-N, the cost is reduced by 64.13% without loss of accuracy compared to the case that every subject takes KLOSCAD-N (the first column in Table 5).

**Fig. 5:**
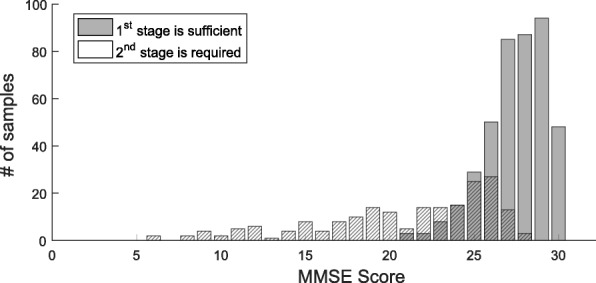
Histogram of MMSE scores. The distribution of the MMSE scores of the test set subjects requiring only first-stage and those requiring two-stages. The two distributions are roughly divided around 25 points, but can not be clearly distinguished only by the MMSE score

Figure 5 is the histogram distribution of the MMSE scores of the test dataset subjects. Subjects that require only first-stage are represented by hatched bars and are represented by shaded bars that require a second-stage. Two groups are roughly divided by point 26, but there are still overlapping parts. The existence of overlapping means that the MMSE score alone can not make a clear diagnosis. In other words, in order to judge whether or not to take the second-stage more clearly, it is necessary to use the designed DNNs.

## Discussion

Comprehensive neuropsychological assessments, in spite of their variety and abundance of information, have not been optimally employed for diagnosing and/or subclassifying dementia by their conventional total and/or subscale scores. In the current study, we developed a low-cost high-accuracy diagnostic framework for diagnosing dementia using a comprehensive neuropsychological battery that includes MMSE. The proposed framework proceeds through four steps: missing data imputation, classifier validation, input variable selection, and two-stage classifications.

Although neuropsychological batteries can provide useful diagnostic information (such as reaction patterns and inter-correlations among them), only overall performance (such as total scores or subscale scores) has been quantified so far in both clinical and research settings. Even if we simultaneously used data from multiple cognitive tests, we could not have improved the diagnostic accuracy for dementia if we had used only the overall performance of each test. For example, Seo et al. [2] proposed the total score of CERAD-N (CERAD-TS), which was a simple sum of multiple cognitive test scores included in the CERAD-N. However, the diagnostic accuracy of the CERAD-TS for dementia was only approximately 3% higher than that of MMSE in a given population.

In our previous work, we showed that the reaction patterns of cognitive tests may provide better performance in diagnostic dementia than simple total scores of the tests [5, 6]. For example, patients with Alzheimer’s showed impaired knowledge-based semantic associations compared with the cognitively normal elderlies who had the same overall performance in the categorical verbal fluency test as the Alzheimer’s disease patients [5]. In addition, we showed that we could improve the diagnostic accuracy for dementia of categorical verbal fluency tests by approximately 10% if we used reaction patterns in the test instead of the total score of the test [6].

Therefore, we may improve the diagnostic accuracy for dementia if we can use the hidden patterns of responses in the multiple cognitive tests included in neuropsychological batteries simultaneously. Data mining approaches have shown remarkable performance in discovering new meaningful information from large datasets and summarizing the information in understandable structure [8]. As we discussed earlier, although a large amount of neuropsychological assessment data have been accumulated, hidden patterns in the data have not been fully analyzed yet. The proposed framework achieved better improvements in diagnostic performance than the CERAD-TS [2] as shown in the fourth column in Table 5. The improvement compared with CERAD-TS was +6.61% for accuracy, 0.044 for AUC, and +0.14 for f1 score.

There were some studies to improve screening accuracy for dementia with MMSE by supplementing other brief cognitive test scores [50] or informant questionnaires [51]. However, it has never been studied whether and how much the supplementation of comprehensive neuropsychological batteries can improve diagnostic accuracy for dementia. To the best of our knowledge, our methodology is the first approach that cascades the screening test (MMSE) and the neuropsychological battery (KLOSCAD-N) for diagnosing dementia.

The proposed framework is effective in three aspects. First, by the proposed two-stage classification approach, 71,570 USD (64.13%) of the cost for 620 subjects was evaluated to be saved without loss of classification performance. Second, through the variable selection step, it was confirmed that only a small amount of KLOSCAD-N variables with 2D-CNN achieved higher performance than the full number of variables. This implies that it is possible to develop more compact assessments with saving time and monetary cost. Third, The proposed framework will be implemented and distributed as a form of software. Non-expert will also be able to obtain additional information about the diagnosis of dementia in addition to the total score by entering the results of the neuropsychological tests into the software. It is expected that the social cost for the overall diagnosis of dementia can be reduced by increasing the usefulness of clinical neuropsychological tests and the possibility of early diagnosis of dementia.

Regarding the limitation of our framework, the diagnosis only focuses on a binary classification problem (normal versus dementia). As for future works, the proposed framework can be extended to a multi-class classification problem such as dementia progress classification (normal versus mild cognition impairment verses dementia) or dementia type classification (Alzheimer’s disease versus vascular dementia versus dementia with Lewy bodies, and so on). However, neuropsychological assessments alone may not be enough to diagnose specific dementia types. In fact, to diagnose the specific dementia types, neuroimaging techniques (MRI and PET) and genetic analysis are performed. Cascading these advanced tests as the next stage of the proposed two-stage classification will further enhance the advantages that we have gained in this study. Another limitation of this study is that the proposed framework cannot explain the hidden patterns learned by DNNs because of the black-box property of deep learning. However, the field of explainable artificial intelligence is being actively studied for visualizing these hidden patterns in nowadays [52]. For the future work, it will be possible to specify meaningful patterns to clinicians through explainable artificial intelligence methodology.

## Conclusion

As validated in the experiments, the proposed framework will contribute to a cost-effective and precise diagnosing of dementia. This effectiveness comes from the introduction of two-stage classification strategy for course-to-fine screening to save the cost. In particular, the improvement of accuracy mainly relies on the elaborate design of a deep learning network using the most recent techniques to fit the best architecture in view of various aspects. In addition to the architecture design of classifier, the missing data imputation, selection of input variables take an important role for the robustness, preciseness, and cost-effectiveness of our framework. The proposed framework could be expanded to a general system for early detection of dementia.

## Abbreviations

AUC: Area under curve
BNT15: 15-item Boston naming test
CERAD-K: Korean version of the consortium to establish a registry for Alzheimer’s disease assessment packet
CERAD-N: Consortium to establish a registry for Alzheimer’s disease assessment neuropsychological battery
CERAD-TS: Total score of CERAD-N
CDR: Clinical dementia rating
CI: Confidence intervals
CLOX: Executive clock drawing
CRT: Constructional recall test
CNE: Cognitively normal elderly
CT: Computerized tomography
CVFT: Categorical verbal fluency test
DSM-IV: Diagnostic and statistical manual of mental disorders
DST: Digit span test
EER: Equal error rate
FAB: Frontal assessment battery
FEAST: Feature selection toolbox
KLOSCAD: Korean longitudinal study on cognitive aging and dementia
KLOSCAD-N: Korean longitudinal study on cognitive aging and dementia neuropsychological battery
kNN: k-nearest neighbor imputation
LLS: Local least squares imputation
MI: Multiple imputation
MINI: Mini international neuropsychiatric interview
MinMax: Minimum-maximum imputation
MMSE: Mini-mental state examination
MRI: Magnetic resonance imaging
RF: Random forest
SVM: Support vector machine
TMT: Trail making test
WLMT: Word list memory test
WLRCT: Word list recognition test
